# Evaluation of the Diagnostic and Prognostic Accuracy of Artificial Intelligence in Endodontic Dentistry: A Comprehensive Review of Literature

**DOI:** 10.1155/2023/7049360

**Published:** 2023-01-31

**Authors:** Mohmed Isaqali Karobari, Abdul Habeeb Adil, Syed Nahid Basheer, Sabari Murugesan, Kamatchi Subramani Savadamoorthi, Mohammed Mustafa, Abdulaziz Abdulwahed, Ahmed A. Almokhatieb

**Affiliations:** ^1^Department of Restorative Dentistry & Endodontics, Faculty of Dentistry, University of Puthisastra, Phnom Penh 12211, Cambodia; ^2^Department of Conservative Dentistry & Endodontics, Saveetha Dental College & Hospitals, Saveetha Institute of Medical and Technical Sciences University, Chennai, 600077 Tamil Nadu, India; ^3^Department of Community Dentistry, School of Dental Sciences, Universiti Sains Malaysia, Health Campus, Kubang Kerian, Kota Bharu, 16150 Kelantan, Malaysia; ^4^Division of operative dentistry, Department of Restorative Dental Sciences, College of Dentistry, Jazan University, Jazan, Saudi Arabia; ^5^Department of Conservative Dental Sciences, College of Dentistry, Prince Sattam Bin Abdulaziz University, P.O. Box 173, Al-Kharj 11942, Saudi Arabia

## Abstract

**Aim:**

This comprehensive review is aimed at evaluating the diagnostic and prognostic accuracy of artificial intelligence in endodontic dentistry.

**Introduction:**

Artificial intelligence (AI) is a relatively new technology that has widespread use in dentistry. The AI technologies have primarily been used in dentistry to diagnose dental diseases, plan treatment, make clinical decisions, and predict the prognosis. AI models like convolutional neural networks (CNN) and artificial neural networks (ANN) have been used in endodontics to study root canal system anatomy, determine working length measurements, detect periapical lesions and root fractures, predict the success of retreatment procedures, and predict the viability of dental pulp stem cells. *Methodology*. The literature was searched in electronic databases such as Google Scholar, Medline, PubMed, Embase, Web of Science, and Scopus, published over the last four decades (January 1980 to September 15, 2021) by using keywords such as artificial intelligence, machine learning, deep learning, application, endodontics, and dentistry.

**Results:**

The preliminary search yielded 2560 articles relevant enough to the paper's purpose. A total of 88 articles met the eligibility criteria. The majority of research on AI application in endodontics has concentrated on tracing apical foramen, verifying the working length, projection of periapical pathologies, root morphologies, and retreatment predictions and discovering the vertical root fractures.

**Conclusion:**

In endodontics, AI displayed accuracy in terms of diagnostic and prognostic evaluations. The use of AI can help enhance the treatment plan, which in turn can lead to an increase in the success rate of endodontic treatment outcomes. The AI is used extensively in endodontics and could help in clinical applications, such as detecting root fractures, periapical pathologies, determining working length, tracing apical foramen, the morphology of root, and disease prediction.

## 1. Introduction

The capacity of an integrated platform to obtain, process, and implement skills and knowledge acquired through education or experience that are usually linked to human intelligence has been defined as artificial intelligence (AI) [1]. AI is a broad idiom that signifies the use of machines and technology to perform tasks similar to those performed by humans. According to Barr and Feigenbaum, AI is a branch of computer science involved with developing innovative application software that exhibits the characteristics users associate with intellectual ability in individual behaviour, such as language comprehension, acquiring knowledge, rationale, problem-solving, and several others [2]. The word AI is generally related to robotics. It explains using technology to create software or a device that quickly imitates human intellectual ability and performs specific tasks [3]. AI has been shown to make it more efficient, accurateness, and specificity in a timely and cost-effective manner, similar to medical professionals [4]. In 1955, a mathematician named John McCarthy proposed the word artificial intelligence, and he is widely regarded as the father of artificial intelligence. He coined the term to describe the ability of machines to perform tasks that can be classified as “intelligent” [5].

**Figure 1:**
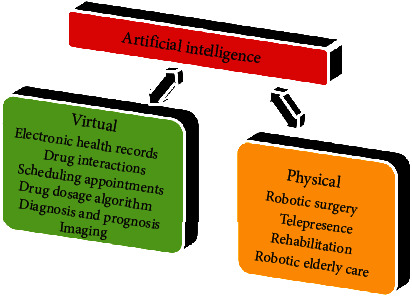
Types of artificial intelligence.

AI is a relatively new technology that has found widespread use in dentistry. AI is primarily made up of an architectural neural network comparable to the brain of humans and imitates human thinking [6]. This structure of neural architecture comprises neurons with robust interconnected systems that primarily function as information systems to determine the problems [7]. The use of neural networks in dentistry has advanced dramatically as science and technology have progressed. The AI technologies have primarily been used in dentistry to diagnose dental diseases, plan treatment, make clinical decisions, and predict the prognosis [8]. There are 2 types of AI in the healthcare sector (Figure 1).

**Figure 2:**
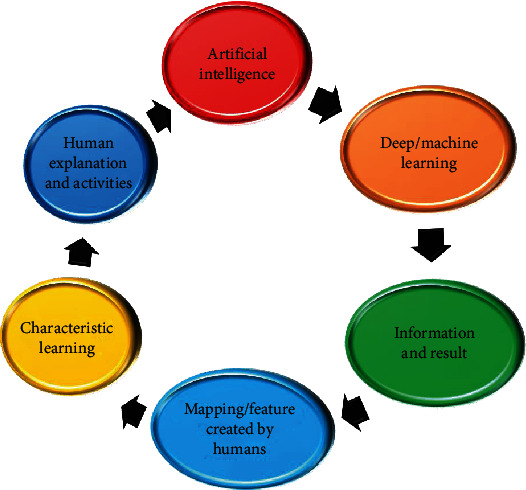
Schematic representation of artificial intelligence model.

AI includes subcategories like machine learning (ML) and related fields like deep learning (DL), advanced analytics, computational linguistics, automation, intelligent agents, and probabilistic reasoning. Machine learning enhances computer-controlled learning without requiring explicit programming. Its main goal is to make automated knowledge acquisition possible without requiring human intervention. AI technologies forecast future occurrences with the current sequence of examples [9]. Figures 2 shows a schematic representation of the AI model.

The purpose of endodontic therapy is to bestow high-quality care to preserve the tooth's function and avoid further complications. In recent years, the diagnosis of root canal pathology, equipment design features, components, and treatment options have advanced dramatically, intending to provide successful endodontic care [8]. The models of AI like convolutional neural networks (CNN) and artificial neural networks (ANN) are being used in the field of endodontics to study the system of root canal anatomy, determine working length measurements, detect periapical lesions and root fractures, predict the success of retreatment procedures, and predict the survival of stem cells in dental pulp [10]. In order to achieve the best results, accuracy in diagnosis and therapeutic judgement is essential. Due to advances in science and technology, many diagnosing devices and therapeutic options have opened up new vistas in diagnostic testing, clinical judgement, and making preparations for the best treatment for root canal system diseases [11]. The AI systems can revolutionize medicine and dentistry by identifying solutions to various clinical problems and making clinicians' jobs easier. Endodontic research has grown in lockstep with other dental specialties [10]. When AI is combined with endodontics, the root canals could be biomechanically prepared with accuracy [12]. The latest advancements in digital applications have also promoted the creation of clinical techniques such as AI-based diagnosis and assisted access cavity preparations to gain easy accessibility to root canals even in obliterated roots [13]. Multiple studies also showed the use of this newly developed concept for endodontic disease diagnosis and treatment planning [8]. From photographic color tooth pictures, Berdouses et al. [14] developed a computer-aided automated approach (ACDS) for the identification of occlusal caries lesions of posterior permanent teeth in accordance with the International Caries Detection and Assessment System (ICDAS II). In order to recognize the distal root shape of the mandibular first molar on panoramic dental radiographs, Hiraiwa et al. [15] used deep learning systems (AlexNet and GoogleNet). Both deep learning algorithms performed diagnostic tasks marginally better than radiologists with extensive training. Based on a random walk segmentation of a graph, a noninvasive differential diagnosis technique for periapical lesions and to pinpoint the precise position of periapical lesions and quantify their volume in CBCT images, a deep CNN-based AI diagnosis model was developed [16]. By extracting relevant features from the matrix, a Random Forest (RF) method is used to classify the picture after segmentation, which results in the formation of a grey level cooccurrence matrix of the image. With an applicable method that can discern gender from maxillary teeth plaster images, automatic gender determination has a success rate of 90% [17]. The facial deformation of a patient after receiving a complete denture prosthesis was predicted using a CAD/CAM (Computer-Aided Design/Computer-Aided Manufacturing) system, which also provided limitations for the digital placement of the complete denture's artificial teeth [18]. Miki et al. [19] investigated a deep convolutional neural network-based automated system for classifying tooth types on dental cone-beam CT images and as a component of an automated dental chart filing system. In addition, Raith et al. [20] introduced an automated computational technique for classifying teeth using artificial neural networks. A superresolution generative adversarial network (SRGAN) was suggested by Moran et al. [21] as a way to generate high-resolution periapical images with a 4-order-of-magnitude improvement. The accuracy of the AI-based diagnosis was identified to be 94.96%, and the level of difficulty for these cases could be decided with high precision in endodontic aspects [22]. The prevalence and usefulness of AI in many activities and applications in dentistry, especially endodontics, have greatly expanded in recent years. AI has the potential to perform diagnostic and prognostic predictions with decision-making ability in the healthcare. The endodontist's knowledge needs to be updated in terms of AI application. This comprehensive review is aimed at evaluating the diagnostic and prognostic accuracy of artificial intelligence in endodontic dentistry.

## 2. Methodology

The literature for this review article was recognized and listed by conducting a thorough search in electronic databases such as Google Scholar, Medline, PubMed, Embase, Web of Science, and Scopus, published over the last four decades (January 1980 to September 15, 2021) by using keywords such as artificial intelligence, machine learning, deep learning, application, endodontics, and dentistry. We were able to find full-length articles. To go through the journals, we used both hand and electronic searching. The information needed for this review was chosen in two stages. The first stage of the articles was chosen based on their titles and abstracts relevant to our research topic. The preliminary search yielded 2560 articles relevant enough to the paper's purpose. 560 articles were removed due to duplication. As a result, we were able to find 2000 articles for the second stage of the selection process.

**Figure 3:**
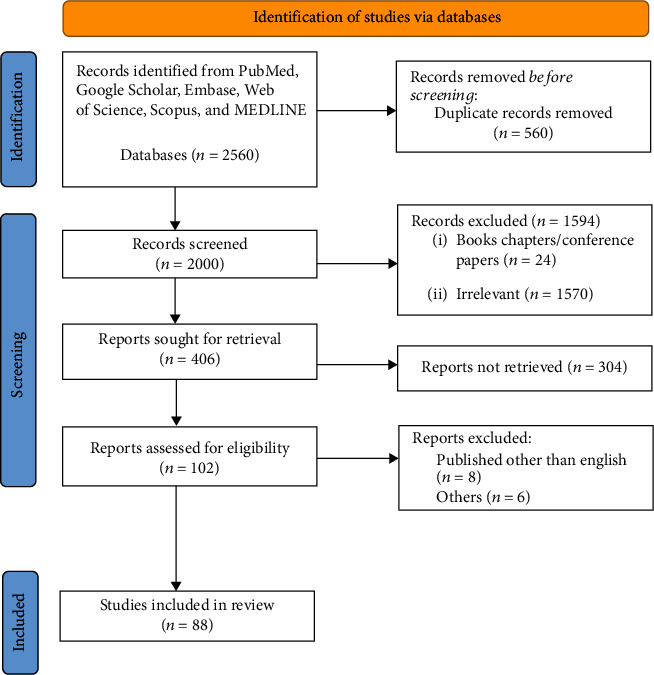
PRISMA flowchart showing the selection process of articles retrieved from different web sources.

The research conducted on AI and its application in endodontics was included in the current review. Further, there must be some predictability or measurable outcomes to be quantified. The articles not related to AI in endodontics, unpublished articles (preprints) available online, articles with only abstract not having the complete text, and articles are written other than the English language were excluded. A total of 88 articles met the eligibility criteria (Figure 3, PRISMA). Before being given to the panel members for critical analysis, the authors' identities and the details of the article were kept hidden. The panel was made up of two members, MIK and AHA. Every article was thoroughly read. The period in which these articles were published was used to track the evolution of AI trends in dentistry and endodontics over time.

## 3. Results

**Figure 4:**
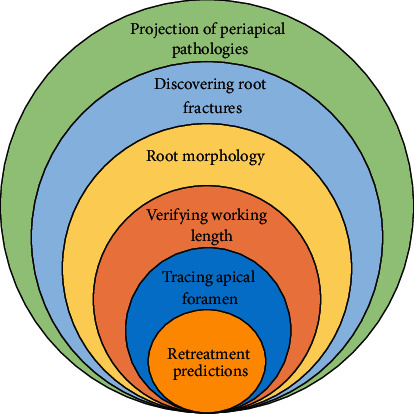
Uses of artificial intelligences in endodontics.

The quantitative data from 66 research articles were analyzed in this comprehensive review. The majority of the studies were conducted in the last two decades. According to the trends, the research on AI in dentistry is gradually increasing. The studies included in the comprehensive review focused primarily on applications of AI in endodontics. The majority of research on AI application in endodontics has concentrated on tracing apical foramen, verifying the working length, projection of periapical pathologies, root morphologies, retreatment predictions, and discovering the vertical root fractures (Figure 4).

## 4. Discussion

The DL with CNN has become the most common AI component used in endodontic diagnostics due to its ability to perform automated lesion segmentation [23]. AI allows multiple and heterogeneous information domains, such as dental history, clinical data, and sociodemographic, to be integrated [9]. AI facilitates scientific investigations by incorporating in silico experimental research options into traditional research setups, supplementing other scientific degrees and existing modelling techniques [24].

### 4.1. Projection of Periapical Pathologies

Apical periodontitis is the most common endodontic disease, accounting for about 75% of jaw lesions that are radiolucent [25]. It is ubiquitous, affecting 33-62% of the population between 20 and 60 years [26]. Panoramic and intraoral periapical radiographs have been the most prevalent 2-D diagnostic aids used during daily clinical settings to recognize apical periodontitis. Radiolucencies are commonly seen in periapical lesions on radiographs. On the other hand, periapical radiographs do not provide reliable information since the actual 3-D anatomy is transformed into a 2-D image [27]. The different types of diagnostic techniques used in endodontics are [13] as follows:

1. Radiography (digital)
2. Cone beam computed tomography (CBCT)
3. Computer-aided diagnosis (CAD)
4. 3D printing
5. Guided endodontics

CBCT imaging, a three-dimensional (3D) imaging technology, is now commonly used in practice by endodontists (about 80%) to diagnose and manage root canal pathologies. When compared to 2D periapical radiography, CBCT scanning has improved periapical pathology detection accuracy [28]. The use of AI technologies to diagnose a periapical pathology from X-rays and CBCT diagnostic tests could aid clinicians in achieving identification precision that is comparable to, if not better than, specialists with good experience [29]. It may also decrease the dentist's diagnostic time and effort by reducing evaluation time by allowing semiautomated documentation [30]. Multiple studies have been done to achieve an accurate diagnosis (Table 1).

### 4.2. Discovering Root Fractures

Vertical root fractures (VRFs) are rare in root canal-treated teeth. VRFs are often insidious since they reveal only minor symptoms and, in many cases, no symptoms at all [31]. VRF is observed to result in 3.7–30.8 percent of root canal-treated teeth, with the mandibular premolars and molars being the most commonly affected [32]. VRF teeth are among the most difficult to treat appropriately, and then, almost all VRF teeth are extracted or treated with hemisection or root separation methods [33]. Early treatment encompassing the resection of infected roots, on the other hand, can result in substantially long survival times for the residual roots, with survival rates of 94% and 64%, respectively, after five and ten years [32]. Initial diagnosis of a VRF will aid to avoid comprehensive tissue injury. The diagnosis of VRF is based on clinical signs and symptoms and radiographic evidence of a fracture line. Moving from conventional radiography to digital imaging and digital image advancement has been attempted to improve the detection ability of radiographic techniques [34]. The diagnosis of a VRF, which could be challenging to detect, is aided by X-ray and CBCT image analysis. Unnecessary surgical procedures or tooth extraction may be required due to a lack of a proper diagnosis. The clinical presentation and the lack of sensitivity of diagnostic imaging in detecting VRFs frequently present a clinician with a diagnosing dilemma [10]. Compared to conventional radiographs, CBCT imaging was good at identifying VRFs in unfilled teeth, while radiographs were slightly better in root-filled teeth. There has been an invitation to explore innovative methods for improving the diagnosis of VRFs due to the incapability of typical techniques to precisely identify VRFs [35]. The AI applications like ML, CNN, and PNN (probabilistic neural network) are used to detect the VRFs [10]. Multiple studies have been reported, which are explained in Table 2.

### 4.3. Root Morphology

A dentist must have a comprehensive understanding of root canal morphology to provide successful root canal therapy. An untreated canal that was possibly missed could result in microbial colonization and, as a result, root canal treatment failure. Given these factors, a dentist wants to possess an absolute understanding of root morphologies and an effective diagnostic tool for identifying them [8]. For a nonsurgical endodontic treatment to be successful, the ability to identify the system of root canal variations is critical. Traditionally, periapical X-rays and CBCT image analysis were used to diagnose this [10]. CBCT for dental use, such as root and canal morphology variants, can now be accurately evaluated in clinics [15]. Even though conventional radiography is still widely used and plays an essential role in root canal pathology, diagnosis, and treatment planning, CBCT provides the highest quality 3D images. As a result, conventional radiograph limitations such as distortion and superimposition of bony and dental structures are no longer an issue [36, 37]. The performance of the DL system of AI in determining the root canal morphology was excellent [15]. The DL system could be helpful in diagnostics, and it classifies images that could aid in understanding images by inexperienced doctors [38, 39]. The ability of the DL algorithm developed by AI and data interpretation to assess the root canal morphologies and its 3-D alterations after instrumentation was demonstrated [40] (Table 3).

### 4.4. Verification of Working Length and Tracing the Apical Foremen

The accuracy of determining the working length is crucial to the success of endodontic treatment [41]. The dental practitioners can master the working length assessment using several different guidelines and techniques, with routine success when different techniques are used [42]. The endodontic treatment necessitates the precise determination of root canal length and the apical foramen. The hand sensation method, radiological determination, and usage of an electronic apex locator are the three methods for measuring root canal length [43]. CBCT and electronic apex locators have recently been used as modern tools for detecting the apical foramen [8, 44]. The electronic apex locator, most frequently used in clinics to measure root canal length, was developed over time using multiple techniques [45]. The root canal treatment prognosis can only be guaranteed when the instrumentation ends at the apical constriction of the root [46]. The ANN diagnosis method helps to improve the diagnosis and results in a better radiographic determination of working length. Further, in a wide range of clinical circumstances, ANNs are used as a judgement system [47]. Few studies have been done by applying artificial intelligence to locate the apical foramen and determine the root canal's working (Table 4).

### 4.5. Retreatment Predictions

In dentistry, the endodontic treatment is successful 90% of the time, with a failure rate of 10%. As a result, a dentist would value the ability to use the AI method to analyze and detect cases falling within this 10% and decide whether extraction or retreatment is preferable [48]. The case-based reasoning (CBR) paradigm was described by Campo et al. [48] to predict nonsurgical endodontic retreatment outcomes and the benefits and risks. In summary, the system determined whether retreatment was necessary. The system incorporates information from regions such as achievement, recollection, and analytical probabilities. The system's power is that it could be able to forecast the outcome of retreatment with reasonable accuracy. The system would only have been as good as the information obtained from the data, which was a limitation.

CBR is the procedure of coming up with answers to problems derived from earlier encounters with similar issues. By recovering similar instances, essential knowledge and information can be incorporated. The problem of variations and the availability of different methods may lead to system heterogeneity [49]. Future research must consider the variability of a human approach, and sample sizes may need to be increased to achieve higher responsiveness, selectivity, and precision [10].

### 4.6. Future Directions

Artificial intelligence has grown in importance as a central concept as we see significant advances in technology and science. Dentists' assessments of patient data are subjective, and research findings have shown that diagnoses are not always consistent among practitioners. Smart, new dental technologies offer a way to improve consistency significantly and, as a result, patient health. Dental research should grow the relationship between oral and general health in the future to concentrate on individualized treatment with patient-centred outcomes. Robotic assistance in dentistry has become possible thanks to technological advancements. “Augmented intelligence” has also been embraced a little too soon in the present scenario. However, the benefits of digital applications will complement human talents and capabilities to provide the best and more cost-effective healthcare to patients. Augmented intelligence based on big data can significantly reduce the number of misdiagnoses and provide more insightful information quickly, accurately, and efficiently. AI can schedule a patient list that includes the patient's ongoing requirements and health information. AI may predict patient-specific drug complications if patient records are made available. AI could help with diagnosis and staging, as well as predict outcomes. This could include things like outcome forecasting or prognostic risk determination.

## 5. Conclusions

In endodontics, AI displayed accuracy in terms of diagnostic and prognostic evaluations. The use of AI can help enhance the treatment plan, which in turn can lead to an increase in the success rate of endodontic treatment outcomes. In recent years, AI has transformed dentistry. It is rapidly progressing, with potential applications spanning various domains such as diagnosis, prognosis, and treatment prediction. The AI is used extensively in endodontics and could help in clinical applications, such as detecting root fractures, periapical pathologies, determining working length, tracing apical foramen, the morphology of root, and disease prediction. However, before integrating AI models into routine clinical work, it is still important to do additional research to test their dependability, relevance, and expenditure.

## Figures and Tables

**Table 1:** Endodontic diagnosis based on AI application.

Author and year	Diagnostic technique	AI method	Accuracy	Reference
Mahmoud et al., 2015	Periapical radiographs	ANN	77.2%	[50]
Hatvani et al., 2018	Dental CT images	CNN	92%	[51]
Ekert et al., 2019	Panoramic radiographs	CNN	85%	[30]
Hiraiwa et al., 2019	CBCT & panoramic radiographs	Deep learning algorithm	86.9%	[15]
Bouchahma et al., 2019	Periapical radiographs	Deep learning algorithm	87%	[52]
Endres et al., 2020	Panoramic radiographs	Deep learning algorithm	72%	[29]
Setzer et al., 2020	CBCT	Deep learning algorithm	93%	[53]
Orhan et al., 2020	CBCT	CNN	92.8%	[54]
Zheng et al., 2020	CBCT	Deep learning algorithm	High	[55]
Pauwels et al., 2021	Periapical radiographs	CNN	83%	[56]

**Table 2:** Detection of vertical root fractures by AI.

Author and year	Diagnostic technique	AI method	Accuracy	Reference
Hassan et al., 2009	CBCT & Periapical radiographs	DICOM 3 Visualization Software	86%	[57]
Varshosaz et al., 2010	CBCT & Periapical Radiographs	ROMEXIS Software	91%	[58]
Metska et al., 2012	CBCT	ACCUITOMO 3D, NEWTOM 3G	93%	[59]
Kositbowornchai et al., 2013	Digital radiographs	PNN	95.7%	[34]
Gunduz et al., 2013	CBCT	ACCUITOMO 3D, VISTASCAN PSP, CCD SENSOR, CONVENTIONAL FILM	Significantly better	[60]
Melo et al., 2013	CBCT	DICOM, DOLPHIN, KDIS3D	73%	[61]
Johari et al., 2017	Periapical radiographs	PNN	96.6%	[62]
Fukuda et al., 2020	Panoramic radiographs	CNN-based detect net with DIGIT version 5	93%	[32]
Vicory et al., 2021	CBCT	ML	Superior	[63]
Xu et al., 2021	CBCT	Pyramids Attention Convolutional Neural Network (FPA-CNN)	Challenging	[64]

**Table 3:** Detection of root canal morphology by AI.

Author and year	Diagnostic technique	AI method	Accuracy	Reference
Hatvani et al., 2018	Dental CT	CNN	Superior	[51]
Hiraiwa et al., 2019	CBCT & panoramic radiography	DL (standard DIGIT algorithm)	86.9%	[15]
Lahoud et al., 2021	CBCT	AI-driven algorithm	High	[65]
Leite et al., 2021	Panoramic radiography	CNN	High	[66]
Başaran et al., 2021	Panoramic radiography	AI-model CranioCatch (deep CNN method)	Promising	[67]
Sherwood et al., 2021	CBCT	DL	Better	[68]
Jeon et al., 2021	Panoramic radiography	CNN-based DL	95.1%	[69]
Zhang et al., 2021	CBCT	DL	High	[70]
Khan et al., 2021	Periapical radiography	DL-based computer vision technique	Better	[71]
Liu et al., 2021	CBCT	CNN	93.3%	[72]

**Table 4:** Application of artificial intelligence for locating the apical foramen and determining the working of the root canal.

Author and year	Diagnostic technique	AI method	Accuracy	Reference
Saghiri et al., 2012	In situ radiographs using Rinn XCP	ANN	93%	[43]
Saghiri et al., 2012	Periapical radiographs	ANN	96%	[73]
Qiao et al., 2020	Circuit system	Neural network model	95%	[45]

## Data Availability

This article includes all types of information used to endorse the review findings.
